# Adenosine Triphosphate Measurement in Deep Sea Using a Microfluidic Device

**DOI:** 10.3390/mi9080370

**Published:** 2018-07-27

**Authors:** Tatsuhiro Fukuba, Takuroh Noguchi, Kei Okamura, Teruo Fujii

**Affiliations:** 1 Japan Agency for Marine-Earth Science and Technology, 2-15 Natsushima–cho, Yokosuka, Kanagawa 237-0061, Japan; 2 Research and Education Faculty, Kochi University, B200 Monobe, Nankoku, Kochi 783-8502, Japan; noguchitk@kochi-u.ac.jp (T.N.); okamurak@kochi-u.ac.jp (K.O.); 3 Institute of Industrial Science, The University of Tokyo, 4-6-1 Komaba, Meguro–ku, 153-8505 Tokyo, Japan; tfujii@iis.u-tokyo.ac.jp

**Keywords:** ATP, microfluidic device, luciferin–luciferase assay

## Abstract

Total ATP (adenosine triphosphate) concentration is a useful biochemical parameter for detecting microbial biomass or biogeochemical activity anomalies in the natural environment. In this study, we describe the development and evaluation of a new version of in situ ATP analyzer improved for the continuous and quantitative determination of ATP in submarine environments. We integrated a transparent microfluidic device containing a microchannel for cell lysis and a channel for the bioluminescence L–L (luciferin–luciferase) assay with a miniature pumping unit and a photometry module for the measurement of the bioluminescence intensity. A heater and a temperature sensor were also included in the system to maintain an optimal temperature for the L–L reaction. In this study, the analyzer was evaluated in deep sea environments, reaching a depth of 200 m using a remotely operated underwater vehicle. We show that the ATP analyzer successfully operated in the deep-sea environment and accurately quantified total ATP within the concentration lower than 5 × 10^−11^ M.

## 1. Introduction

The importance of organic and inorganic matter circulating globally and locally in ocean environments has prompted research in the field of marine environmental microbiology on the abundance, distribution and roles of oceanic microbes represented by *Eubacteria* and *Archaea*. Microbes have relevant roles especially in submarine hydrothermal sites or hydrocarbon seepage areas, because they support unique ecosystems as primary producers [1]. Even with the rapid progress of sophisticated DNA and RNA analysis methodologies, the determination of the number of microbial cells in seawater samples is still indispensable for estimating microbial biomass and for studying their spatiotemporal distribution. Generally, microscopic or flow-cytometric counting of fluorescently stained or genetically labeled cells are conducted by well-trained researchers in onboard or onshore laboratories, using samples collected during scientific cruises [2]. However, the quantitative determination of microbial ATP (adenosine triphosphate), which is a ubiquitous biomolecule utilized for energy conversion and storage in living cells, in seawater has been regarded as one of the most useful alternatives to labor-intensive microscopic cell enumerations [3,4]. In particular, the quantity of particulate ATP (pATP) in seawater is a representative proxy of the microbial biomass in a sample [5]. Since dissolved ATP (dATP)—an important carbon and phosphorus source for marine microbes—is also related to microbial activity [6], the sum of pATP and dATP (total ATP (tATP)) is a useful parameter indicative of the presence of biogeochemical events, such as submarine volcanisms [1], hydrocarbon seepages [7] and occasional supply of organic resources (e.g., whale falls) [8]. By realizing a compact and portable apparatus for in situ ATP quantification, it becomes possible to analyze the distribution of microbial biomass anomalies with an unprecedented spatiotemporal resolution, which enables an efficient exploration of the underwater resources as well as deep sea environmental and microbiological studies [9].

**Figure 1:**
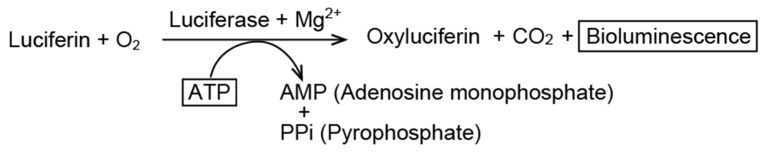
Schematic of luciferin–luciferase reaction for ATP quantification.

ATP concentration can be determined by the L–L (luciferin–luciferase) bioluminescence assay [10] (see Figure 1) that is a simple method for ATP quantification generally used for hygiene monitoring [11,12]. The microfluidic technology has been applied to automate flow analyses in various fields, including biochemistry and it has been used in marine environments for microbial gene analysis [13], nutrient analysis [14] and trace metal analysis [15,16]. Previously, we developed and evaluated microfluidic devices and in situ analyzers for intermittent (non-continuous) quantitative determination of ATP based on the L–L assay [17,18,19]. For the further improvement of the spatiotemporal resolution of the in-situ measurement, a new system with continuous measurement capability was developed and evaluated for practical oceanography applications [20,21,22]. In these studies, two microfluidic devices with single function for microbial cell lysis and bioluminescence detection were used in combination. In this study, we improve the new system by merging the single function microfluidic devices into one device and its performance in the deep-sea environments is examined by comparing the in-situ ATP quantification results with manually processed values.

## 2. Materials and Methods

### 2.1. In situ ATP Analyzer

**Figure 2:**
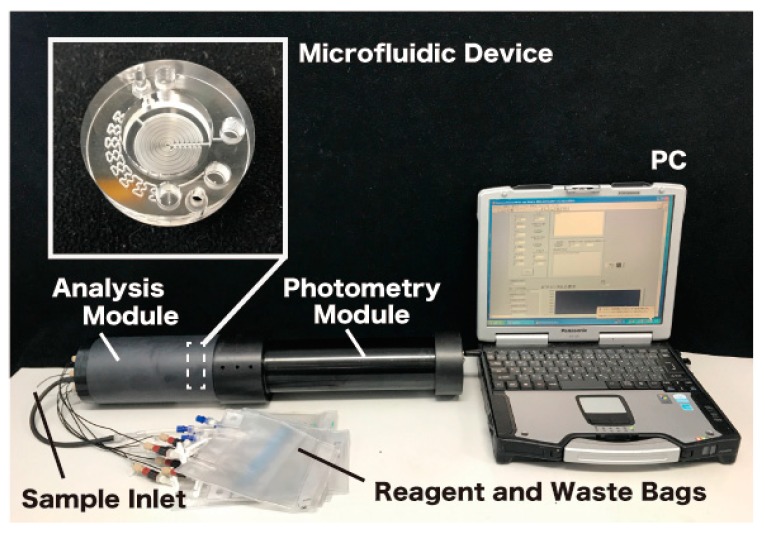
The in-situ adenosine triphosphate (ATP) analyzer and the microfluidic device developed and evaluated in this study with a laptop PC for real-time control and data acquisition.

The in-situ ATP analyzer that we have produced can quantify the ATP contained in seawater continuously at a depth of 3000 m. Since the seawater samples are not filtered before the analysis, tATP concentrations are measured. The analyzer consists of an analysis module, which is the core component and of a photometry module for the bioluminescence intensity measurements based on the L–L reaction (see Figure 2).

**Figure 3:**
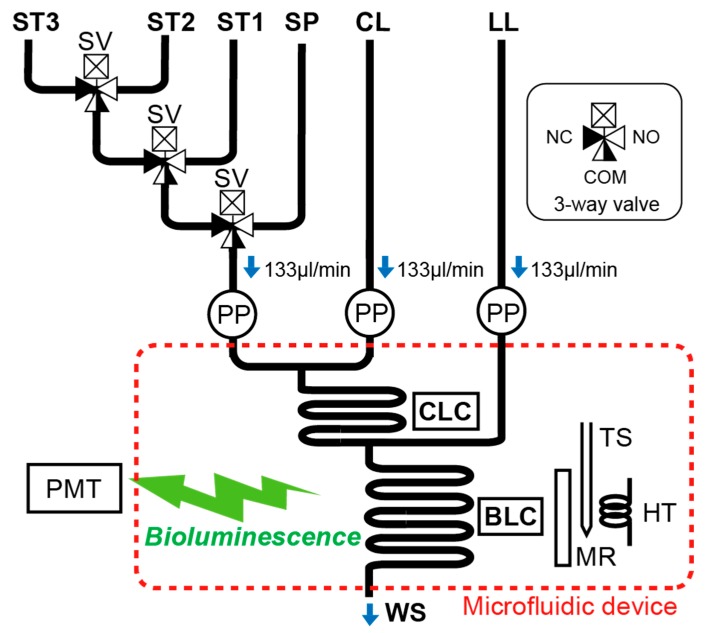
Flow diagram of the in-situ ATP analyzer. ST1–3: ATP standard solutions 1–3, SP: sample, CL: cell lysis reagent, LL: L–L reagent, CLC: cell lysis channel, BLC: bioluminescence channel, WS: waste outlet, PP: peristaltic pump, SV: solenoid three-way valve, TS: temperature sensor, HT: heater, MR: mirror, PMT: photomultiplier tube.

**Figure 4:**
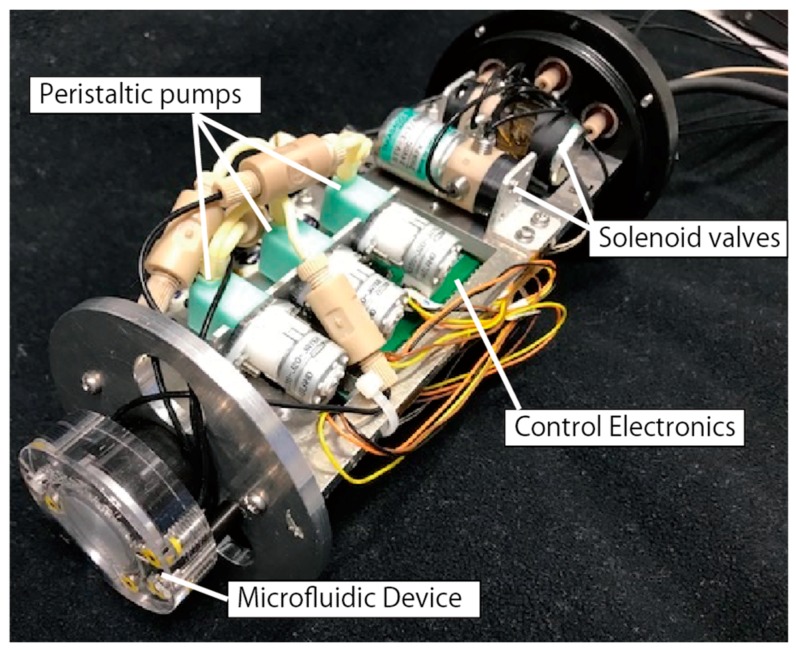
The analysis module for the in-situ ATP analyzer. Three peristaltic pumps and three solenoid-actuated three-way valves were employed as the pumping device.

**Figure 5:**
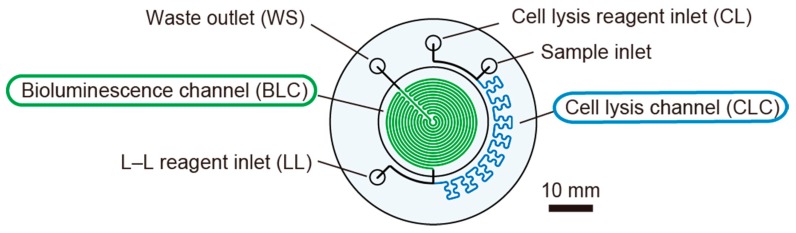
Microfluidic device for the in-situ ATP analyzer.

A flow diagram of the ATP analyzer is shown in Figure 3. The analysis module (see Figure 4) is connected to the microfluidic device for the L–L assay (Figure 2 and Figure 5) and contains three miniature peristaltic pumps (RP-0.15S-P15A-DC5VS, Aquatech Co., Ltd., Daito, Japan), three solenoid-actuated three-way valves (STV-3-1, Takasago Electric, Inc., Nagoya, Japan) and the control electronics. The fluidic components are connected using black-colored Teflon^TM^ FEP (fluorinated ethylene propylene) tubes (1519, IDEX Health & Science, Oak Harbor, WA, USA) to shield the analysis module from the ambient light. A cascaded connection of the three-way valves enables the selection of four kinds of fluids (the sample and three standard solutions) through the three valves. The control electronics is based on a miniature microprocessor board (ML100 series, Microtec Co., Ltd., Funabashi, Japan) and is used to control the valves, pumps and heater. Sequential scenarios for pump and valve operation and temperature setting can be stored on a micro SD card in the control electronics. All the components of the analysis module are enclosed in a cylindrical plastic container filled with fluorinated oil (Fluorinert FC-43, 3M, Maplewood, MN, USA) for electrical insulation and pressure equalization during underwater operations. Since it employs a pressure-balanced configuration, the system does not require a complicated and large pumping mechanism to manage the elevated underwater hydrostatic pressure and overcome the differences between the internal and external pressures. The seawater sample is led from the outside of the system through a short tube to the container. The chemicals for the L–L assay and standard solutions are stored in plastic bags, connected to the system via the Teflon^TM^ FEP tube. One end of the oil-filled analysis module has a transparent window facing the photometry module. The total power consumption of the in-situ ATP analyzer is approximately 24 W in maximum (when all the valves and the heater are activated) including the photometry module described later.

### 2.2. Microfluidic Device

The microfluidic device for the in-situ ATP analyzer is made of transparent PMMA (Poly (methyl methacrylate)) to detect the bioluminescence produced by the L–L reaction taking place in the device (Figure 5). Channels for cell lysis and the L–L reaction are engraved by means of precision milling on a PMMA disk (47 mm in diameter and 2 mm in thickness) and bonded with the top plate (11 mm in thickness) with 1/4-28 UNF threaded tubing interfaces by a diffusion bonding method (Takasago Electric., Inc., Nagoya, Japan). Each channel is 0.5 mm wide and 1.5 mm deep. First, the sample (SP) is mixed with a cell lysis (CL) reagent that releases ATP from the microbial cells as the mixture passes through a serpentine cell lysis channel (CLC), which is approximately 133 mm in length (this takes approximately 23 s). The extracted ATP is mixed with the L–L reagent immediately at the end of the CLC to initiate the L–L bioluminescence reaction that takes place in the serpentine bioluminescence channel (BLC) folded in a circular shape. Along the approximately 482 mm of the BLC, bioluminescence is emitted and its intensity corresponds to the ATP concentration in the sample. Even though the microfluidic device does not include a mixer structure, the asynchronous pulsated flow generated by the three peristaltic pumps enables the mixing of the reagents. The mixing ratio of the sample and reagents is 1:1:1 (SP/CL/LL). At the flow rate of 133 µL/min for each component, it takes approximately 54 s for the final mixture to pass through the L–L reaction channel. The waste is discharged from a waste port (WS) and collected in a plastic bag outside of the analysis module. A miniature flat mirror (MR) reflects the bioluminescence to a PMT (photomultiplier tube) in the photometry module. A film heater (HT) and a temperature sensor (TS) are fixed behind the mirror in order to maintain the optimal temperature for the L–L reaction. The TS was placed just behind the HT and the activation of the HT was regulated by the control electronics in the analysis module. The temperature is typically kept to 35 °C (higher than room-temperature) considering the situation of operation under the room-temperature condition with the waste heat generation from pumps and valves in the analysis module.

### 2.3. Photometry Module

The photometry module consists of a photon-counting PMT and a data logging electronics located in a cylindrical pressure-tight housing. In contrast to the analysis module that has a pressure-balanced configuration, all the key components of the photometry module are protected from ambient hydrostatic pressure. The PMT faces a pressure-resistant glass window (18 mm in thickness) fixed at the end of the pressure-tight housing for the bioluminescence measurement. The measured bioluminescence intensity data are stored on a micro SD card in the data logging electronics based on ML100 series (Microtec Co., Ltd., Funabashi, Japan) and transferred in real-time to a PC connected to the in-situ ATP analyzer via RS-232 format.

### 2.4. Reagents

For the L–L assay, a commercially available kit for bacterial biomass determination (CheckLite HS Set, Kikkoman Biochemifa Co., Tokyo, Japan) containing the ATP releasing (cell lysis) reagent and the L–L reagent was modified for seawater sample measurement [18,19]. EDTA (ethylenediaminetetraacetic acid, Wako Pure Chemical Industries, Ltd., Osaka, Japan) was added to the ATP releasing reagent at a final concentration of 10 mM to avoid precipitation in the presence of the seawater samples or the seawater-based ATP standard solutions. To avoid the adsorption of the reagents and the adhesion of natural particles or debris to the micro-channels, 2% (*v*/*v*) Tween 20 (MP Biomedicals LLC, Santa Ana, CA, USA) was added to the L–L reagent. Both EDTA and Tween 20 were sterilized by autoclaving prior to use to eliminate potentially contaminating ATP. Seawater-based ATP standard solutions (5 × 10^−12^, 5 × 10^−11^, 5 × 10^−10^ M ATP and blank) were prepared from an original ATP standard solution (2 × 10^−6^ M ATP, Kikkoman Biochemifa Co., Tokyo, Japan) by diluting it with autoclaved artificial seawater (Daigo’s artificial seawater SP for marine microalgae medium, Nihon Pharmaceutical Co., Ltd., Tokyo, Japan). All reagents and standards were aseptically introduced into sterilized plastic bags (DSF-300, Tsukada Medical Research Co. Ltd., Ueda, Japan) for use in the in-situ analyzer.

### 2.5. Evaluations

The in-situ ATP analyzer was evaluated in the laboratory environment using three ATP standard solutions and blank to acquire the relationship between ATP concentration and bioluminescence intensity. For the evaluation, ATP standard solutions and blank were filled in an aseptic plastic test tube and introduced into the analyzer from the sample inlet. After the saturation of the bioluminescence intensity at each ATP concentration, consecutive 10 s values were used for calibration.

### 2.6. In Situ Evaluations

**Figure 6:**
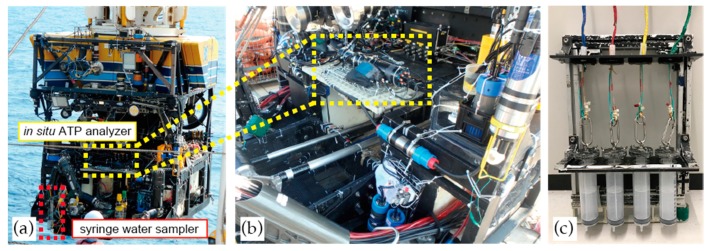
Remotely operated vehicle (ROV) HYPER-DOLPHIN with the in-situ ATP analyzer (**a**); Close-up view of the in-situ ATP analyzer mounted on the payload space of the ROV (**b**); The syringe water sampler (**c**).

The evaluation of the in-situ ATP analyzer developed in this study was carried out in the real field during the scientific cruise KS-17-J07C using R/V SHINSEI MARU and ROV (remotely operated vehicle) HYPER-DOLPHIN (Japan Agency for Marine-Earth Science and Technology, JAMSTEC) in May 2017. The in-situ ATP analyzer was mounted on the ROV (see Figure 6), which provided electricity and RS-232 communication. Real-time monitoring of the bioluminescence data and control of the in-situ ATP analyzer were carried out on board using a PC, which was connected to the ROV via an underwater cable. During the 2021st dive of HYPER-DOLPHIN, the ROV and the analyzer were dived to the Oomuro Hole located in the northern Izu-Ogasawara arc, where the existence of hydrothermal activity has been reported [23]. Continuous tATP measurements were performed from the sea surface to the seafloor at a depth of approximately 200 m. The system calibration was also carried out in situ using the ATP standard solutions to compensate the changes on the reagent flow-rate or temperature on the microfluidic device caused by unexpected effect of elevated hydrostatic pressure on the pumps, temperature sensor and heater controller. Furthermore, effect of the temperature and pressure on the L–L reaction [24] must be considered for accurate in situ quantification of tATP. Water samplers (see Figure 6c) equipped with aseptic plastic syringes were installed on the ROV for sample collection for data comparison. The collected water samples were transferred to clean test tubes immediately after the dive and tATP concentration was measured by a conventional method using test tubes on board using a desktop luminometer (NU-2600, Microtec Co., Ltd., Funabashi, Japan) and a CheckLite HS Set (Kikkoman Biochemifa Co., Tokyo, Japan) without modification. Bioluminescence intensity was integrated for 10 s and all the measurements were triplicated.

## 3. Results

**Figure 7:**
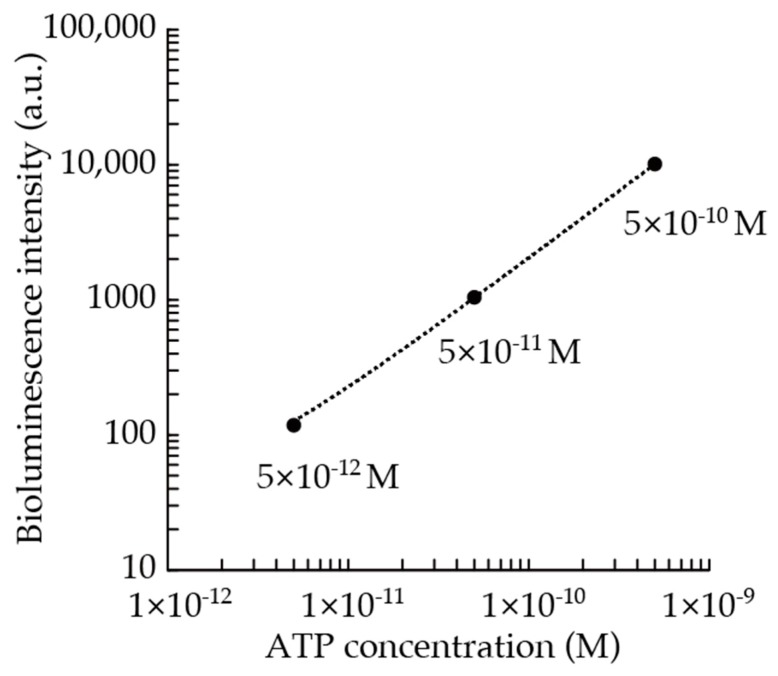
Relationship between ATP concentration and bioluminescence intensity measured by the in-situ ATP analyzer in the laboratory environment. Continuous data for 10 s at each ATP concentration were integrated, the blank value was subtracted and plotted with the ATP concentration.

As a result of the calibration in the laboratory environment, highly linear relationship (*R*^2^ > 0.99) between the ATP concentration and the bioluminescence intensity was obtained (Figure 7). Therefore, extrapolation of the result of in situ calibrations obtained using the 5 × 10^−12^, 5 × 10^−11^ M of ATP standards and the blank is reasonable up to 5 × 10^−10^ M of ATP concentration.

**Figure 8:**
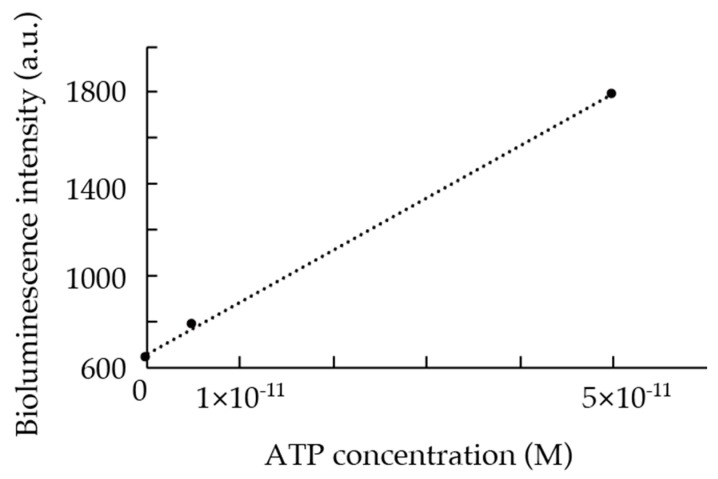
Relationship between ATP concentration and bioluminescence intensity measured by the in-situ ATP analyzer during the 2021st dive of HYPER-DOLPHIN. Consecutive 10 s measurements at each ATP concentration were integrated and plotted with the ATP concentration.

**Figure 9:**
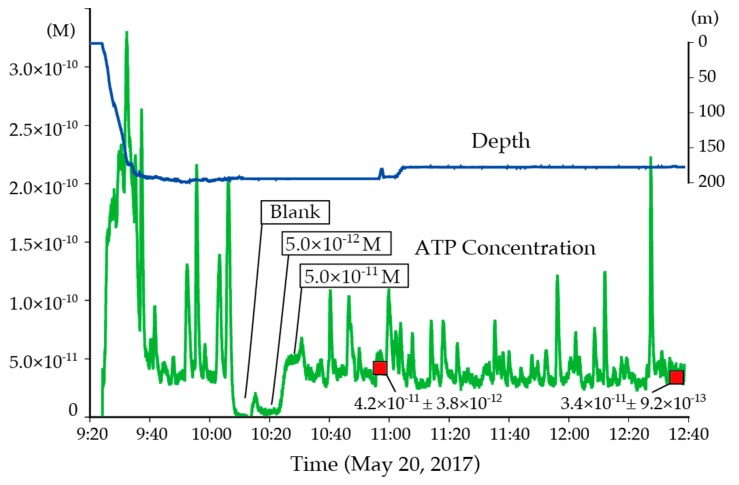
Result of in situ ATP measurement at the Oomuro Hole during the 2021st dive of HYPER-DOLPHIN. Raw bioluminescence data were converted to ATP concentration and plotted as the green line with time. The time was shifted 3 min ahead considering the time-lag between sample intake and bioluminescence emission in the analyzer. The depth profile measured by a depth sensor on the ROV is shown in the blue line. ATP concentrations measured onboard using the collected water samples were shown as red squares.

A continuous tATP measurement from the sea surface to the bottom of the Oomuro Hole area was carried out successfully using the in-situ ATP analyzer developed in this study. After calibrating the system using ATP standard solutions, a linear correlation between the ATP concentration and the bioluminescence intensity (*R*^2^ > 0.99) was obtained (see Figure 8) and was later applied to the measured raw data to calculate tATP concentration in the samples (see Figure 9). For data exceeding the bioluminescence intensity corresponding to the ATP concentration of 5.0 × 10^−11^ M, such as data from the surface, the calibration formula was extrapolated.

At the beginning of the measurement at the surface, the measured tATP concentration was extraordinarily low and increased rapidly within five minutes. This was due to a time lag at the beginning of the measurement required to reach and fill the reagents and the sample to the microfluidic device. A high concentration of tATP, corresponding to 1.0 × 10^−10^ M or more, was measured at the surface after the reagents and sample were filled in the microfluidic device. This is consistent with the formation of a larger microbial biomass layer at the surface supported by photosynthetic primary productions. As the ROV dived more deeply, the tATP concentration decreased rapidly. In situ calibration was successfully performed at the bottom of the sea, as shown in Figure 9. The measurement precision rates of the analyzer, estimated from the 3σ value calculated from three consecutive 10 s measurements of bioluminescence intensity of the 5.0 × 10^−12^ and 5.0 × 10^−11^ M ATP standard solutions, were 43% and 4.5% (2.2 × 10^−12^ and 2.3 × 10^−12^ M) of the measured values, respectively. Conversely, the measurement precision rate calculated for manually measured triplicate data were 14% and 9.9% of the measured values. The in-situ ATP analyzer showed better performance for the determination of ATP concentration close to 5 × 10^−11^ M. In contrast, the measurement precision rate for lower ATP concentrations was better for the desktop apparatus. This was due to fluctuations of the bioluminescence intensity data during the measurement of the 5 × 10^−12^ M standard.

In the Oomuro Hole, tATP concentration was in the range of 2.0 to 3.0 × 10^−11^ M with occasional increases to values higher than 1.0 × 10^−10^ M. The ATP concentrations measured on board of two seawater samples collected by the ROV were consistent with the data provided by the in-situ ATP analyzer, as shown in Figure 9. The occasional ATP concentration peaks were likely due to the introduction of inorganic-organic aggregated particles including microbes [25] or marine snow particles originated from the surface water. After more than 2 h of the operation of the in-situ ATP analyzer was halted because of an electric trouble alert on the ROV system.

## 4. Discussion

In this study, an in-situ ATP analyzer was developed by employing a PMMA microfluidic device as a core element of the system. The performance of the in-situ ATP analyzer was evaluated in a real deep-sea environment. The ATP analyzer successfully measured the tATP concentrations at different depths, providing data that were consistent with those measured manually. These results demonstrate that a portable, simple and reliable flow analysis system such as our microfluidic device can be used in extreme environments for real-time biochemical analyses. The calculated measurement precision rates showed successful value (4.5%) at 5 × 10^−11^ M range of ATP concentration and decreased performance for the determination of ATP concentrations as low as 5 × 10^−12^ M (43%). Because the lower measurement precision has been led by fluctuation of light intensity value and it is caused by electric noise from control electronics, improvement in the precision may be achieved by the reducing the noise by enhancing the control electronics in the near future.

The pATP concentration required for the quantitative estimation of the microbial biomass can be determined using the current system by subtracting dATP concentration, measured by the L–L assay without using the cell lysis reagent, from the tATP concentration [26]. However, to measure the dATP, it is necessary to perform additional calibrations of the in-situ ATP analyzer, specific for dATP measurements. In the current system, the ATP measurement must be interrupted during the system calibration with ATP standard solutions. The calibration process required approximately 30 min during the in-situ evaluation at the Oomuro Hole. To overcome this limitation, we have been developing and evaluating a novel calibration method using an optically activated caged ATP as an internal standard [21,22,26]. By applying the new calibration technology, it will be possible to utilize the in-situ ATP analyzer for practical underwater resources surveys and environmental assessment missions in the near future.

Instruments for in situ flow-analysis based on the technologies developed in this study and consisting of a simple microfluidic device and a pumping apparatus can be employed for various in situ biological and biochemical analyses in human-inaccessible extreme environments.
